# Aggregative responses of marine predators to a pulsed resource

**DOI:** 10.1111/1365-2656.14214

**Published:** 2024-11-15

**Authors:** Gail K. Davoren, Laurie D. Maynard, Kelsey F. Johnson, Paloma C. Carvalho, Julia Gulka, Edward Jenkins, Lauren M. Lescure, Emily Runnells, Ashley Tripp

**Affiliations:** ^1^ Department of Biological Sciences University of Manitoba Winnipeg Manitoba Canada

**Keywords:** capelin, forage fish, foraging ecology, *Mallotus villosus*, Newfoundland, predator–prey interactions, resource pulse, seabird

## Abstract

1. Pulsed resources resulting from animal migrations represent important, transient influxes of high resource availability into recipient communities. The ability of predators to respond and exploit these large increases in background resource availability, however, may be constrained when the timing and magnitude of the resource pulse vary across years.
2. In coastal Newfoundland, Canada, we studied aggregative responses of multiple seabird predators to the annual inshore pulse of a key forage fish species, capelin (*Mallotus villosus*).
3. Seabird aggregative responses to fish biomass were quantified from weekly hydroacoustic and seabird surveys during July–August within an annually persistent foraging area (10 km^2^) associated with a cluster of capelin spawning sites across 10 years (2009–2010, 2012, 2014–2020). Seabird predators included breeding members of the families Alcidae (Common Murres *Uria aalge*, Razorbills *Alca torda*, Atlantic Puffins *Fratercula arctica*) and Laridae (Great Black‐backed Gulls *Larus marinus*, American Herring Gulls *L. argentatus smithsonianus*) and Northern Gannets *Morus bassanus*, along with non‐breeding, moulting members of the Family Procellariidae (Sooty Shearwaters *Ardenna griseus*, Great Shearwaters *A. gravis*).
4. The inshore migration of spawning capelin resulted in 5–619 times (mean ± SE, 146 ± 59 times) increase in coastal fish biomass along with a shift towards more, larger and denser fish shoals. Within years, seabird abundance did not increase with inshore fish biomass but rather peaked near the first day of spawning, suggesting that seabirds primarily respond to the seasonal resource influx rather than short‐term variation in fish biomass. Across years, the magnitude of the seabird aggregative response was lower during low‐magnitude resource pulse years, suggesting that predators are unable to perceive low‐magnitude pulses, avoid foraging under high competitor densities, and/or shift dietary reliance away from capelin under these conditions. The seabird response magnitude, however, was higher when the resource pulse was delayed relative to the long‐term average, suggesting that predators increase exploitation during years of minimal overlap between the resource pulse and energetically demanding periods (e.g. breeding, moulting).
5. This long‐term study quantifying responses of multiple predators to a pulsed resource illustrates the ability of natural systems to tolerate natural and human‐induced disturbance events.

## Graphical abstract

Aggregative responses of multiple marine predators (seabirds) to a pulsed resource subsidy (fish) are quantified across 10 years. Unlike previous studies, predator abundance did not vary with resource biomass but rather peaked upon pulse initiation, suggesting that predators primarily respond to pulsed resource influxes rather than short‐term variation in resource biomass.

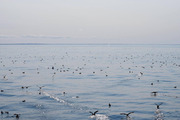

## 1 INTRODUCTION

Natural communities are dynamic and have characteristic disturbance regimes, with frequency, scale and intensity specific to each system (Odum et al., 1995). One type of disturbance that alters communities is pulsed resources, defined as infrequent and transient events where superabundant resources become temporarily available (Ostfeld & Keesing, 2000; Yang et al., 2008, 2010), which occur in many terrestrial (e.g. synchronous seed/fruit production; Ostfeld & Keesing, 2000) and aquatic ecosystems (e.g. phytoplankton blooms; Yang et al., 2008, 2010). On an annual scale, the occurrence of a pulsed resource may be predictable when seasonally abundant pulses of allochthonous subsidies result from animal migrations (‘migration‐mediated subsidies’; Bauer & Hoye, 2014). These seasonally predictable pulses are common in aquatic ecosystems (Bauer & Hoye, 2014; Willson & Womble, 2006), especially from spawning migrations of marine or anadromous fish (Flecker et al., 2010). In these cases, consumer species in the recipient community likely have evolved strategies to efficiently exploit this seasonally predictable increase in background resource availability; however, their efficiency in exploiting the resource pulse may be constrained by variation in the fine‐scale timing of the pulse within a season as well as variation in the magnitude of the pulse among years (Yang et al., 2008, 2010). Indeed, although the occurrence of spawning fish migrations is seasonally predictable, the timing can vary due to environmental conditions experienced prior to or during migration (Wright & Trippel, 2009) and the inter‐annual magnitude of the resource pulse can vary with environmental factors that influence recruitment and year‐class strength. Overall, this variation provides natural experimental conditions to investigate short‐term behavioural responses of consumer species (e.g. aggregative response) that may lead to altered species interactions and community structure (e.g. species composition and relative abundances; Ostfeld & Keesing, 2000; Yang et al., 2008, 2010).

In the Newfoundland and Labrador Shelf marine ecosystem, off the east coast of Canada, a key forage fish species, capelin (*Mallotus villosus*), undergoes extensive (>250 km) annual migrations from offshore wintering areas to coastal regions to spawn (Carscadden et al., 2013). High abundances of spawning capelin migrating into coastal regions act as an annually predictable pulsed subsidy, bringing large influxes of energy and nutrients from offshore regions. Capelin is an important prey species for many marine predators in coastal Newfoundland during the summer, including breeding seabirds (Carscadden et al., 2002; Davoren, 2024), non‐breeding seabirds (Carvalho & Davoren, 2019, 2020) and migrating baleen whales (e.g. humpback whale *Megaptera novaeangliae*; Johnson & Davoren, 2021a). Behavioural responses by predators to the inshore arrival of spawning capelin include shifts in foraging behaviour (Davoren, 2024), such as tactics used to locate prey (e.g. local enhancement and memory, Bairos‐Novak et al., 2015), foraging location and effort (Gulka et al., 2019; Gulka & Davoren, 2019; Lescure et al., 2023; Maynard et al., 2021), as well as species interactions (Carvalho et al., 2020; Carvalho & Davoren, 2020; Maynard et al., 2019). Additionally, the distributional and density patterns of spawning capelin during the summer often shape those of predators in coastal regions (Carvalho & Davoren, 2019; Davoren, 2007, 2013a; Johnson & Davoren, 2021b; Whitehead & Carscadden, 1985). Nonlinear functional relationships between predator and capelin densities also have been illustrated (Piatt, 1990; Piatt & Methven, 1992), similar to aggregative responses of marine predators and prey in other regions (e.g. Cox et al., 2013; Marston et al., 2002; Piatt et al., 2007; Sigler et al., 2004).

Despite these predator responses to the inshore migration of spawning capelin, few studies have examined the response of multiple predator species simultaneously to inter‐annual variation in the timing and magnitude of the seasonal pulse of spawning capelin in coastal Newfoundland. A better understanding of these predator responses is a key management priority, as the Newfoundland and Labrador capelin stock (Northwest Atlantic Fisheries Organization, NAFO, Divisions 2J3KL) collapsed in the early 1990s, and remains in a collapsed state (Buren et al., 2014, 2019). This stock collapse was associated with persistent changes in capelin biology and behaviour, including southerly shifts in the species distribution, a 3‐week delay in the timing of spawning and changes in life history characteristics (e.g. smaller, younger spawning fish; Buren et al., 2019). Despite the collapsed state, capelin remain locally abundant at and nearby coastal spawning sites in July and August, where predators form large multi‐species feeding aggregations (Davoren, 2007, 2013a). Research in the last few decades has shown high interannual variation in the timing of capelin spawning and biomass at these sites (Crook et al., 2017; Davoren, 2024; Davoren et al., 2012), which have been linked to species‐specific behavioural responses by predators (Berard & Davoren, 2020; Carvalho & Davoren, 2020; Davoren, 2024; Garthe et al., 2011; Gulka et al., 2019, 2020; Lescure et al., 2023). Divergent responses across predators likely result from differences in breeding status, dietary reliance on capelin and diet flexibility and the diverse suite of foraging strategies, resulting in varying mobility and foraging constraints (Yang et al., 2010).

Here, we investigate the aggregative responses of multiple seabird predator species to the resource pulsed subsidy during the summer in coastal Newfoundland represented by the inshore spawning migration of capelin across 10 years (2009–2010, 2012, 2014–2020). We estimated fish biomass during ship‐based hydroacoustic surveys within a known fine‐scale (10 km^2^) predator foraging hotspot (Davoren, 2013a) to quantify the timing and magnitude of this resource pulse within years. During these surveys, we simultaneously estimated the abundance of multiple breeding and non‐breeding seabird predators to quantify their aggregative response to this resource pulse within years. We predicted a positive, nonlinear increase in seabird abundance with increasing capelin biomass within years and that responses would be guild‐specific as multiple foraging guilds are represented (i.e., wing‐propelled pursuit‐divers, plunge‐divers, surface feeders). We also tested the hypothesis that the annual magnitude and timing of the resource pulse influences the guild‐specific magnitude of seabird aggregative response. Following the quantitative framework of Yang et al. (2010), the magnitude of the resource pulse in each year was calculated by dividing the natural logarithm of fish biomass in the survey with the highest (peak) biomass (Rpeak) by the fish biomass in the survey with the lowest (baseline) biomass (Rbaseline). The magnitude of the consumer response in each year was calculated similarly (ln Cp/Cb). We predicted that a higher magnitude resource pulse will result in higher magnitude predator aggregative responses (Yang et al., 2010), as posited by foraging theory (Stephens & Krebs, 1986). We also predicted that inter‐annual variability in the timing of the resource pulse will result in lower magnitude responses, as predators become less able to track changes in prey biomass (Willson & Womble, 2006; Yang et al., 2010). Overall, by quantifying seabird predator responses to the inshore pulse of spawning capelin in coastal Newfoundland, we aimed to determine the influence of varying timing and magnitude of capelin biomass on seabird foraging efficiency, thereby providing an important basis for ecosystem‐based fisheries management.

## 2 MATERIALS AND METHODS

### 2.1 Study area

**FIGURE 1:**
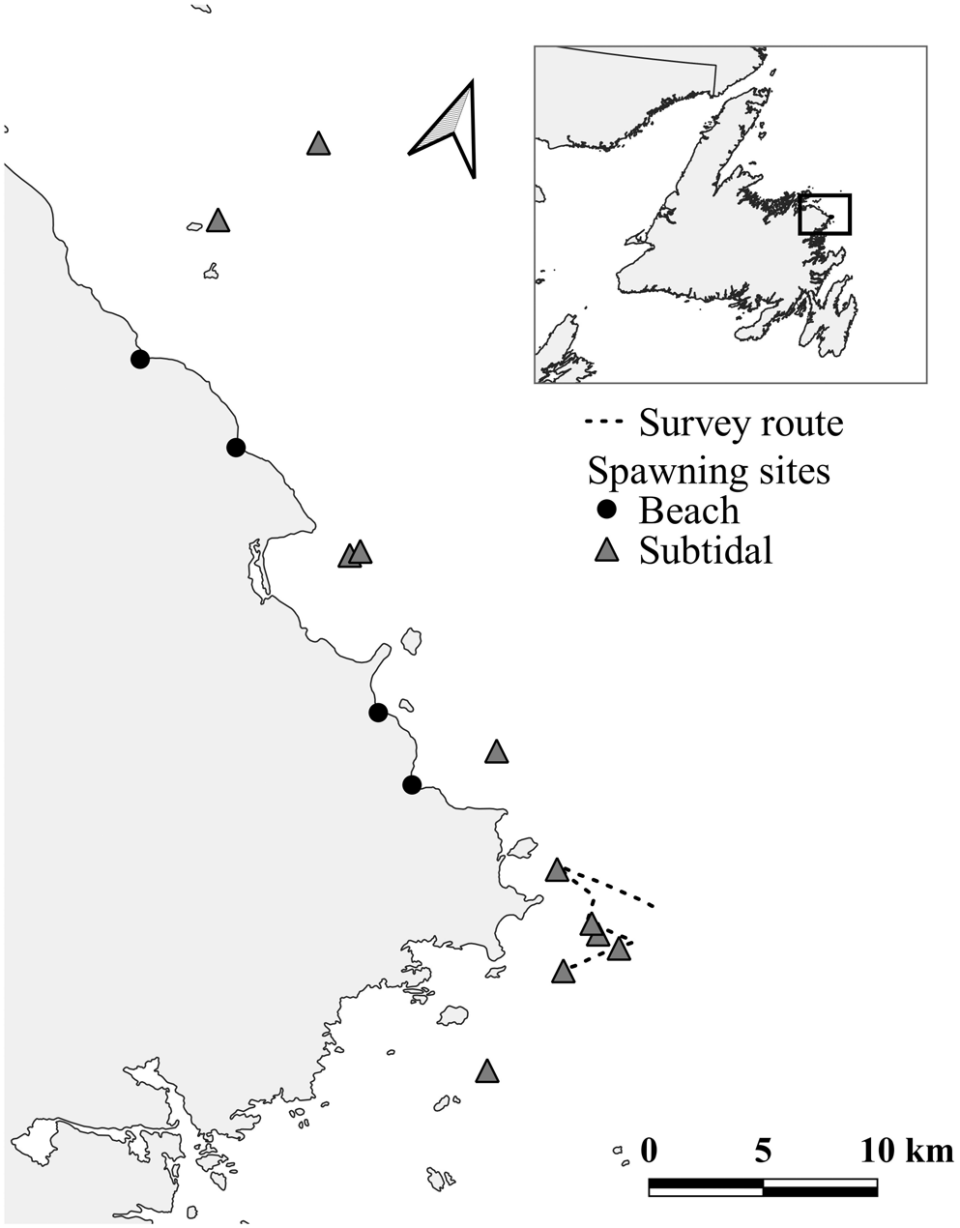
Location of the study region (square) on the northeast coast of Newfoundland, Canada, and the survey area, indicating the cluster of four persistently used subtidal capelin spawning sites (triangle), along with other subtidal (triangle) and beach (intertidal; circle) spawning sites, as well as the survey route (dashed line).

On the northeast Newfoundland coast, an annually persistent multi‐species biological hotspot (10 km^2^), including multiple seabird and baleen whale species (Davoren, 2007, 2013a), forms over a cluster of four persistently used subtidal capelin spawning sites in shallow water (15–40 m; Penton & Davoren, 2012; Figure 1). Within this predictable predator foraging area, capelin aggregations can be predictably found once capelin has arrived inshore to spawn (Davoren et al., 2003a, 2006). Numerically dominant predators include locally breeding and migratory non‐breeding seabirds (Davoren, 2007, 2013a, 2024). Within the larger study area, there are a number of seabird breeding colonies, including Funk Island, which supports the largest colony of Common Murres *Uria aalge* (~472,259 breeding pairs, b.p.; Wilhelm et al., 2015) and the fourth largest and most oceanic colony of Northern Gannets *Morus bassanus* (9987 b.p.; Chardine et al., 2013) in the northwest Atlantic. Several smaller multi‐species breeding colonies occur within 20 km of the coast, including the largest breeding colony of Atlantic Puffins *Fratercula arctica* (16,859 b.p.) on the northeast Newfoundland coast (James Island), which also supports 2757 b.p. of Razorbills *Alca torda* (Environment and Climate Change Canada, ECCC, unpublished data). Great Black‐backed Gulls *Larus marinus* and American Herring Gulls *L. argentatus smithsonianus* also breed in small numbers (10–50 b.p.) on most seabird colonies as well as larger (50–100 b.p.) species‐specific colonies on separate islands in the study area (ECCC, unpublished data; Maynard & Davoren, 2020). Additionally, Great Shearwaters *Ardenna gravis* and Sooty Shearwaters *A. grisea* migrate from South Atlantic breeding grounds (Brooke, 2004; Hedd et al., 2012) and arrive at the study area in high abundance during July to moult (Carvalho et al., 2022; Davoren, 2013a). These seabird species represent a number of foraging guilds, including wing‐propelled pursuit‐divers of the Family Alcidae (i.e. Common Murres, Razorbills, Atlantic Puffins; hereafter ‘alcids’) and the Family Procellariidae (Great and Sooty shearwaters; hereafter ‘shearwaters’), plunge‐divers (i.e. Northern Gannets) and surface‐feeders of the Family Laridae (i.e. Great Black‐backed Gulls, American Herring Gulls; hereafter ‘gulls’). As predators are expected to only respond to the portion of the local biomass that is available for consumption (Guillemette et al., 2018), it is important to note that capelin in the survey area were within ingestible size ranges (<180 mm; Maxner et al., 2016) and found at accessible depths (<50 m) for all predators considered in this study.

### 2.2 Data collection

A fine‐scale (16–20 km) survey was conducted within the biological hotspot (Davoren, 2007, 2013a; Figure 1) approximately weekly throughout July–August over 10 years (2009–2010, 2012, 2014–2020). The survey followed a zig‐zag design to include each of the four annually persistent capelin spawning sites (Figure 1) and varied in length among years due to logistical limitations (2009–2010: 21.3 ± 0.4 km; 2012: 16.4 ± 5.4 km; 2014–2020: 13.7 ± 3.0 km). Surveys were conducted at a constant speed (9–11 km h^−1^) during daylight aboard a 11–13 m commercial fishing vessel.

During surveys, a standard strip transect method was used to record seabird counts (Method I, Tasker et al., 1984), whereby continuous counts of all seabird species were made by one or two observers from the bridge (~2 m above sea level) in a 90° arc from the bow out to 300 m on the port side of the vessel. This strip width was determined to be reasonable during early surveys in the study area (2000–2003; Davoren, 2007, 2013a), as the detection probability of birds on the water declined beyond this width, as observed in other studies (Barbraud & Thiebot, 2009). Surveys were only conducted under good conditions for animal detectability, including low sea state (<2 m swells), high visibility (5–10 km) and low wind speed (<35 km h^−1^). The number of individuals of each species, and behavioural descriptions (foraging, on water, flying and flight direction) were recorded in addition to latitude, longitude and time of each count (Birds and Beasty Counter version 1.0, 1998; D. Senciall, Fisheries and Oceans Canada, St. John's, Canada).

Throughout each survey, high‐resolution hydroacoustic data were recorded continuously using a BioSonics DTX 6000 scientific hydroacoustic system (BioSonics, Inc., Seattle, Washington). The sounder was operated through a 70 kHz split‐beam transducer (15° beam angle) calibrated with a tungsten carbide sphere (Foote et al., 1987) and mounted in a towed body. The transducer was towed on the starboard side of the vessel ~1 m below the surface and, thus, acoustic signals were not reliable until ~3 m. The sounder was operated at 1 ping s^−1^ and pulse duration of 0.4 ms. Raw high‐resolution hydroacoustic data (*s*_v_, volume backscattering coefficients) were continuously acquired above a threshold of −90 dB.

We also monitored capelin presence and spawning activity in the study area during regular visits to known capelin intertidal (beach) spawning sites (every 1–2 days) and subtidal spawning sites (every 3–7 days; see detailed methods in Crook et al., 2017). We quantified the approximate timing of capelin inshore arrival as the first day spawning capelin were present at any monitored spawning sites. The timing of spawning was defined as the first day capelin eggs in early developmental stages, indicating fertilization in the past 1–2 days (Fridgeirsson, 1976), were found adhered to sediment at these sites.

### 2.3 Data processing

Echoview software (version 4, SonarData, Myriax Software Pty. Ltd., Hobart, Tasmania) was used to analyse the hydroacoustic data. Acoustic signals within 0.5 m of the seabed were first omitted if the seafloor could not be distinguished from biology (e.g. side‐lobing; Simmonds & MacLennan, 2005). To quantify acoustic biomass (area backscattering coefficient, or *s*_a_, m^2^ m^−2^; MacLennan et al., 2002), we integrated acoustic signals using a minimum *s*_v_ threshold of −80 dB in 100 m segments along the survey. This threshold allowed the detection of single capelin targets in this shallow area (15–50 m), while filtering out most other noise. Acoustic signals are primarily due to capelin nearby the spawning sites (Davoren et al., 2006), although sand lance (*Ammodytes* spp.) is often present at one of the subtidal spawning sites and low densities are detected acoustically (Morrison & Davoren, 2024). We used a published target strength–length relationship for capelin (Rose, 1998) to convert *s*_a_ into number of fish m^−2^ (i.e. density) and applied the average length and mass of capelin captured at the spawning sites in each year to convert density into capelin biomass (g m^−2^).

Seabird counts were merged with capelin biomass within each 100 m segment along each survey. Although all air‐breathing marine predators were counted during surveys, we focused analyses on the numerically dominant seabirds (see Davoren, 2007, 2013a, 2024), which included breeding seabirds (alcids, Northern Gannets, gulls), as well as non‐breeding seabirds (shearwaters; Davoren, 2007, 2013a). We further focussed the analysis on birds that were more likely to be foraging by excluding counts of flying seabirds for species/groups that do not likely forage during flight (i.e., alcids, shearwaters; Davoren, 2013a). During the surveys, alcid species included primarily Common Murres (96.6%), but also Razorbills (0.5%) and Atlantic Puffins (2.8%). Due to inter‐annual variation in the number of each shearwater species (Carvalho & Davoren, 2019), Sooty and Great shearwaters were grouped (Davoren, 2013a). Counts of Northern Gannets and large gull species on the water and flying within the strip transect were included in the analysis as both groups forage during flight and birds on water may suggest recent foraging locations (Good, 2020; Mowbray, 2020; Weseloh et al., 2020). Gull species included American Herring Gulls (83.1%) and Great Black‐backed Gulls (16.9%) and were grouped for analysis. The total number of birds per survey (i.e. sum of all 100 m segments) was calculated for each seabird species/group to quantify four seabird variables per survey: the total number of alcids (pursuit‐divers), large gulls (surface‐feeders), Northern Gannets (plunge‐divers) and shearwaters (pursuit‐divers).

As variation in prey characteristics other than biomass have been shown to influence predator responses (Benoit‐Bird et al., 2011, 2013; Johnson & Davoren, 2021b; Maniscalco et al., 1998; Ostrand et al., 1998; Sigler et al., 2017), we also quantified shoal characteristics for each survey. As this process was time‐consuming, we only quantified shoal characteristics across the first 7 years of surveys (2009–2010, 2012, 2014–2017). For each survey, fish shoals were identified by visually assessing each echogram in Echoview software, following Johnson and Davoren (2021b). The majority (97%) of shoals observed were identified as capelin based on capelin‐likely s_v_ thresholds at different depths, along with the distinctive shape of capelin shoals (see Davoren et al., 2006), indicating that few other shoaling species were encountered in the survey area. Shoals were not included if prey were scattered in low density along the surface, which were often combined with bubble trails from birds (Benoit‐Bird et al., 2011). Due to the dominance of capelin in the survey area, two characteristics of each capelin shoal were estimated following Davoren et al. (2006). First, we measured the maximum horizontal (width, m) and vertical (height, m) length of each shoal and then estimated the two‐dimensional area of each shoal by multiplying the height by the width. Second, the acoustic biomass (area backscattering coefficient, or *s*_a_, m^2^ m^−2^; MacLennan et al., 2002) of the shoal was determined by integrating acoustic signals using a higher minimum *s*_v_ threshold of −70 dB to better define the prey shoal. Although depth is an important shoal characteristic because it reflects prey accessibility for air‐breathing predators (e.g. Ostrand et al., 1998; Womble et al., 2014), this was not included as a characteristic because data exploration revealed that >90% of shoals were accessible within 5–10 m from the ocean surface due to the shallow nature of the surveyed area (15–50 m). As capelin shoals could be predictably found in the survey area once capelin arrived inshore to spawn (Davoren et al., 2003a, 2006), shoal persistence on a fine scale (~1 km) was not measured, as in other studies (Sigler et al., 2017). Most shoals (>95%) matched previous descriptions of ephemeral shoals moving toward, away and between spawning sites (see Davoren et al., 2006) but a few each year matched previous descriptions of large, stationary spawning shoals (see Davoren et al., 2006) that could be predictably found on a fine scale (<1 km) at discrete spawning sites across 1–2 weeks. Overall, the following four prey variables were quantified for each survey: mean fish biomass (g m^−2^) over the survey (i.e. average capelin biomass over all 100 m segments along the survey, ‘survey biomass’), mean capelin biomass (g m^−2^) within shoals (‘shoal biomass’), mean area of capelin shoals (m^2^; ‘shoal area’) and the number of shoals per survey. Survey biomass was calculated for surveys in all years of this study (2009–2010, 2012, 2014–2020), while shoal characteristics were only calculated for the first 7 years (2009–2010, 2012, 2014–2017).

### 2.4 Statistical analysis

Statistical analyses were performed using the R version 4.2.1 software (R Development Core Team, 2023), unless otherwise indicated. Prior to statistical analysis, all prey and seabird variables were log (*x* + 1) transformed to meet the underlying assumptions of parametric statistics. Means are presented as ±SE and due to the high variability of prey biomass and seabird abundance among surveys, we explored trends where *p* < 0.1.

To quantify the timing and magnitude of the capelin resource pulse within years, generalized additive mixed models (GAMMs) were developed where the response variables were the four prey variables [log(*x* + 1) transformed] from each survey and the smoothed parametric predictors included the difference (in days) of each survey from before (negative) or after (positive) the first date of capelin spawning within the study area (‘spawning timing’; Figure 1). Year and the total number of 100 m survey segments within each survey, indicating survey effort, were included as random intercepts in all models to account for the effects of variation among years and survey length. For all models, predictors were smoothed using cubic regression splines and if the estimated degrees of freedom (edf) equaled one, suggesting that the relationship was linear (Wood, 2006), the predictor was converted to parametric and the model was run again. Models were run using restricted maximum likelihood and the package *mgcv* (Wood, 2018). We also calculated the Pearson's correlation coefficients and associated *p*‐values between each of our four prey variables (survey biomass [g m^−2^], shoal biomass [g m^−2^], shoal area [m^2^], total number of shoals) across the 7 years (2009–2010, 2012, 2014–2017) all variables were measured.

To evaluate the response of different seabird foraging guilds to shifts in the coastal prey field and the timing of this shift within years, we developed GAMMs, similar to the process described above, where the response variables were the four seabird variables [log(*x* + 1) transformed] from each survey. Smoothed parametric predictors included log(*x* + 1)‐transformed survey biomass (g m^−2^) and spawning timing, along with year and the total number of 100 m survey segments within each survey (i.e. survey effort) as random intercepts. These two parametric predictors were used as both spanned all 10 years of surveys and were not correlated with each other but were correlated with other prey variables and, thus, represented general changes in the prey field (Figure 3). Pearson correlation coefficients were also calculated between each of the four seabird variables (total numbers of alcids, shearwaters, gulls, Northern Gannets per survey).

To test whether inter‐annual variation in the magnitude and timing of the resource pulse influenced the magnitude of the predator response, we calculated the magnitude of the resource pulse as the natural log of fish biomass during the survey with the highest (or peak) biomass (Rp) divided by the fish biomass in the survey with the lowest (or baseline) biomass (Rb) within a year (ln Rp/Rb), following Yang et al. (2010). The magnitude of the consumer response was calculated in the same manner (ln Cp/Cb; Yang et al., 2010) for each seabird species/group. ANCOVAs were run in JMP Pro (version 16.2; SAS Institute, North Carolina, USA) to compare the magnitude of the resource pulse (ln Rp/Rb) and timing of the pulse (date of Rp) to the magnitude of the predator response (ln Cp/Cb) among seabird species/groups, where the slope of this relationship indicated the consumer response magnitude relative to the resource pulse magnitude (Yang et al., 2010). When using ANCOVAs, we first tested the null hypothesis of equal slopes between seabird species/groups (i.e. no interaction between ln Rp/Rb and seabird species/group) and, if this null hypothesis was not rejected, we then tested the null hypothesis of equal mean seabird species/group abundance adjusted for ln Rp/Rb (Quinn & Keough, 2002).

### 2.5 Ethics Statement

All research activities were conducted in accordance with the Canadian Council on Animal Care (CCAC) and approved by the University of Manitoba Fort Garry Campus Animal Care Committee (Protocol Numbers: F08‐022, F12‐020, F16‐017, F20‐017).

## 3 RESULTS

**FIGURE 2:**
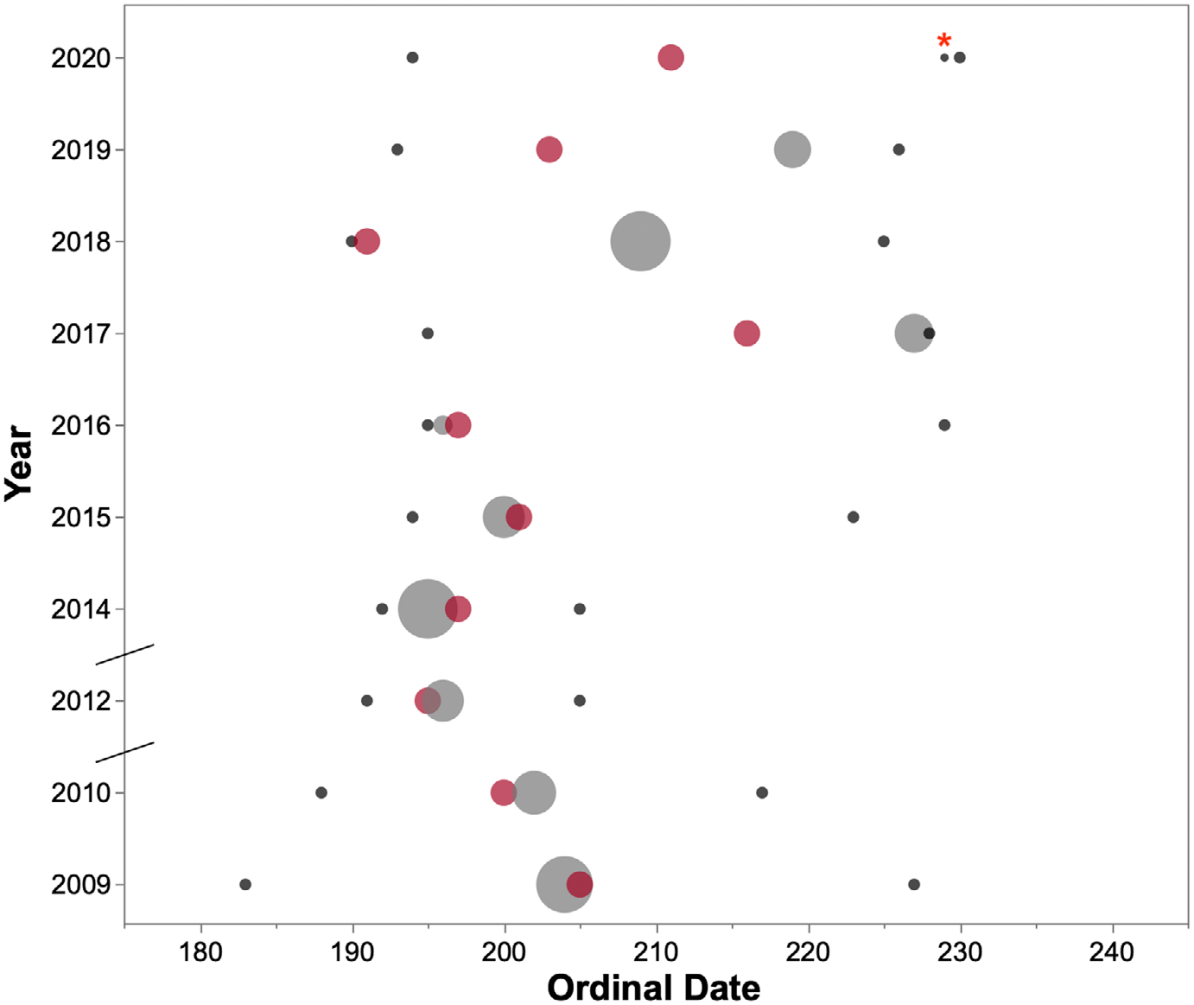
The timing of annual peak capelin biomass (grey circles), with circle size indicating the annual peak survey biomass (g m^2^; see Table 1 for peak survey biomass values), along with the date of the first and last hydroacoustic survey in each year (small black circles) and the date capelin spawning began (red circles). The ordinal date is on the *x*‐axis, where January 1 = Day 1 in each year. In 2020, the peak capelin biomass is indicated with “*” because it was the lowest biomass on record and was difficult to visualize. Breaks in the *y*‐axis indicate where sampling years were noncontinuous.

The first date of capelin spawning varied inter‐annually by up to 25 days within the study period (Table 1). Peak capelin biomass varied among years in the surveyed area, being highest during 2009, 2014 and 2018 (0.230–0.259 g m^−2^), moderate during 2010, 2012, 2015, 2017 and 2019 (0.098–0.138 g m^−2^), and lowest during 2016 (0.027 g m^−2^) and 2020 (0.005 g m^−2^; Table 1). The date of first spawning or arrival in the study area was correlated with the date of peak capelin biomass (*R*_8_ = 0.683, *p* = 0.042), but this relationship appeared to weaken from 2017 to 2020 (Table 1; Figure 2).

**TABLE 1:** Summary of the number, mean length and date range of surveys conducted during July–August in each year (2009–2010, 2012, 2014–2020) over a cluster of annually persistent subtidal capelin spawning sites on the northeast coast of Newfoundland, Canada, along with the date capelin began spawning at these sites, surveys with the peak (Rp) and baseline (Rb) capelin biomass (g m^−2^) in each year (magnitude of the pulsed resource is ln Rp/Rb and indicated in parentheses), date of the survey with peak capelin biomass, surveys with the peak (Cp) and baseline (Cb) total number of birds per km in each year (magnitude of the bird response is ln Cp/Cb and indicated in parentheses), and the dates of peak number of total birds (number of days predator abundance peaked after capelin biomass peaked indicated in parentheses, with negative numbers indicating that predator abundance peaked before the date of peak capelin biomass).

Year	No. surveys	Survey length (km)	Survey date range	First date of spawning	Peak/baseline survey biomass (g m^−2^) (ln Rp/Rb)	Date of peak survey biomass	Peak/baseline no. birds/km (ln Cp/Cb)	Date of peak no. birds/km
2009	10	21.7 ± 1.3	Jul 2–Aug 15	Jul 24^a^	0.230/0.001 (5.4)	Jul 23	40.9/0.3 (4.8)	Jul 28 (6)
2010	9	21.9 ± 0.8	Jul 7–Aug 8	Jul 19	0.138/0.001 (4.9)	Jul 21	272.5/8.8 (3.4)	Jul 21 (0)
2012	7	16.4 ± 0.5	Jul 9–23	Jul 13	0.125/0.005 (3.2)	Jul 14	71.0/20.3 (1.2)	Jul 16 (2)
2014	4	14.6 ± 0.3	Jul 11–24	Jul 16	0.254/0.02 (2.5)	Jul 14	198.3/8.7 (3.1)	Jul 18 (4)
2015	6	11.2 ± 1.8	Jul 13–Aug 11	Jul 20	0.126/0.002 (4.1)	Jul 20	27.3/5.2 (1.7)	Aug 5 (17)
2016	3	13.4 ± 0.4	Jul 14–Aug 16	Jul 15^b^	0.027/0.005 (1.7)	Jul 14	66.6/21.8 (1.1)	Aug 3 (20)
2017	5	12.0 ± 0.3	Jul 14–Aug 15	Aug 4	0.109/0.0008 (4.9)	Aug 15	308.2/8.4 (3.6)	Aug 9 (−6)
2018	6	13.8 ± 0.3	Jul 9–Aug 7	Jul 10	0.239/0.0004 (6.4)	Jul 28	51.3/7.1 (2.0)	Aug 8 (11)
2019	6	13.3 ± 0.3	Jul 12–Aug 14	Jul 22	0.098/0.0005 (5.3)	Aug 7	127.8/5.5 (3.1)	Jul 22 (−16)
2020	6	13.3 ± 0.3	July 12–Aug 17	Jul 29^b^	0.005/0.00002 (3.2)	Aug 17	27.7/4.8 (1.8)	Aug 5 (14)

^a^ Indicates the timing of capelin spawning at regularly monitored intertidal sites in the larger study region in a year (2009) when capelin did not spawn at the cluster of subtidal sites.

^b^ Indicates capelin arrival in the survey area in 2 years (2016, 2020) when spawning did not occur at regularly monitored intertidal and subtidal spawning areas in the larger study region.

### 3.1 Prey field shifts

**FIGURE 3:**
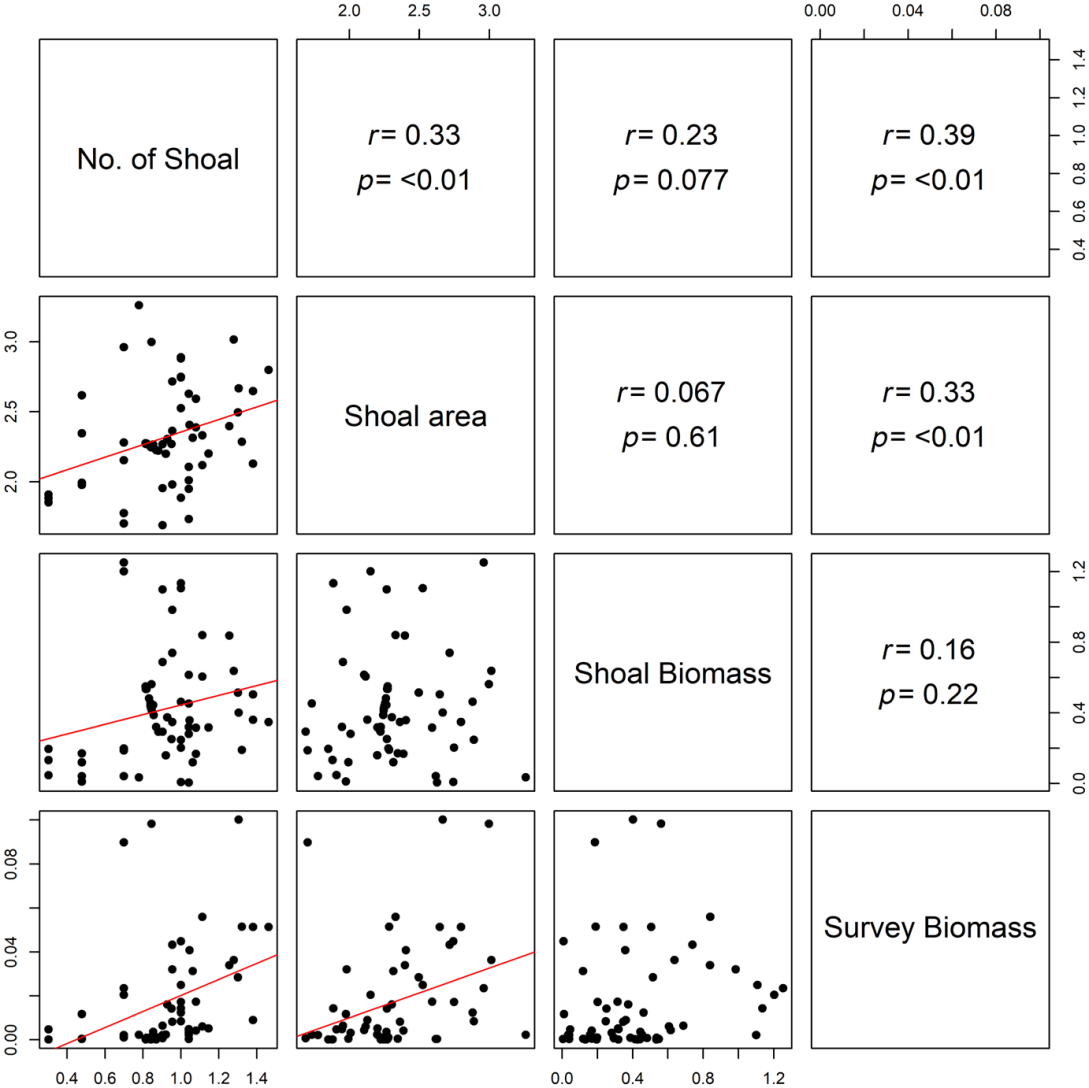
Pair‐wise relationships between prey variables (survey biomass [g m^−2^], shoal biomass [g m^−2^], shoal area [m^2^], total number of shoals) per survey during approximately weekly surveys over a cluster of subtidal spawning sites of capelin on the northeast coast of Newfoundland, Canada during July–August, 2009–2010, 2012, 2014–2017. Lower panels show relationships graphically, with red lines indicating significant relationships (*p* < 0.1) and upper panels show *r* and *p* values from Pearson correlation tests.

**FIGURE 4:**
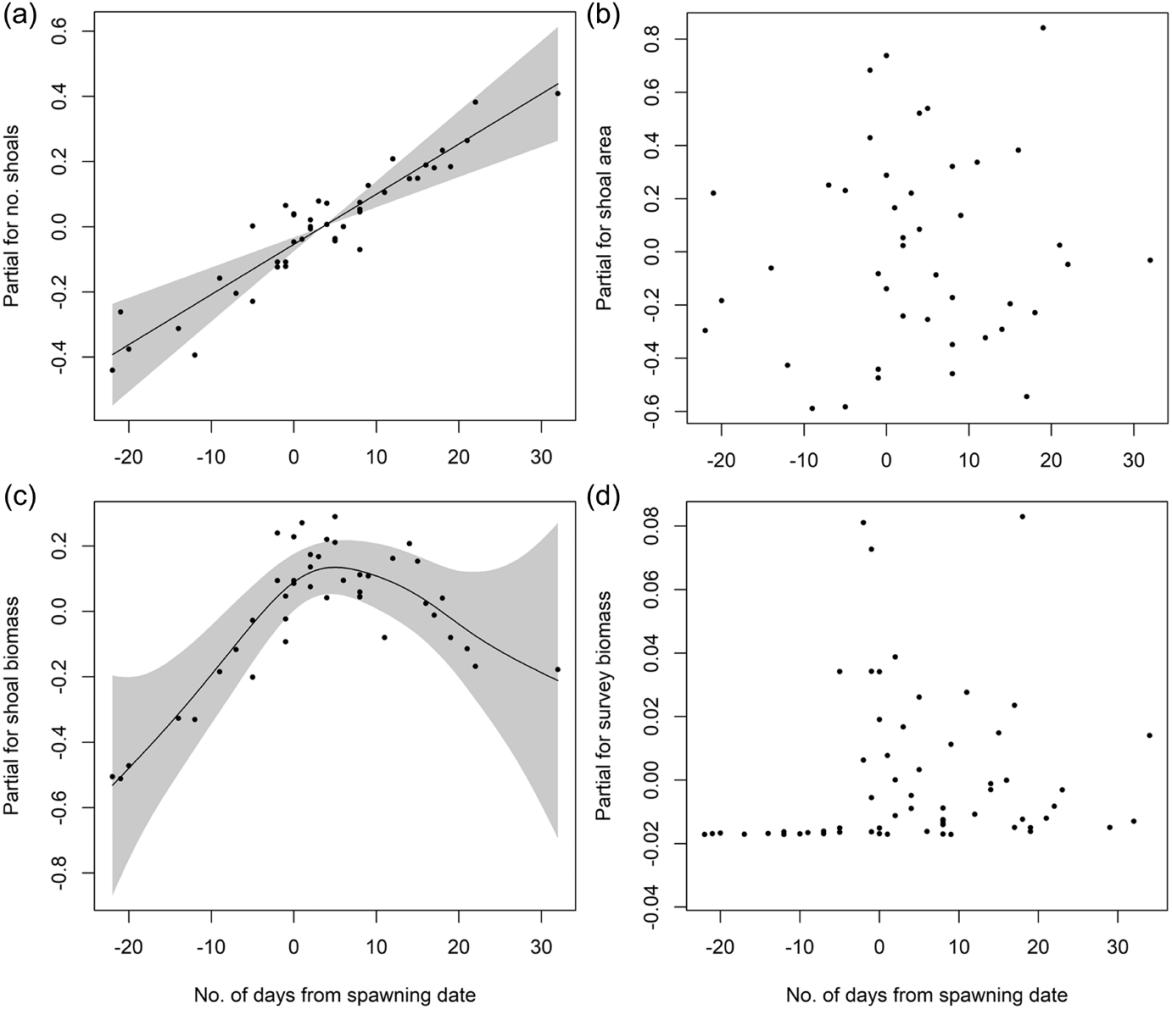
Estimated effect of the timing of capelin spawning (zero represents the first day of spawning in a particular year, while negative numbers represent days before spawning and positive numbers represent days after spawning) on (a) total number of shoals (b) shoal area, (c) shoal biomass and (d) survey biomass on the northeast coast of Newfoundland, Canada during July–August, 2009–2010, 2012, 2014–2017. Grey area represents 95% confidence intervals. Note that the analysis for survey biomass included surveys in all years of this study (2009–2010, 2012, 2014–2020), but for number of shoals, shoal area and shoal biomass only years up to 2017 were included in the analysis, because these prey variables were not measured from surveys during 2018–2020.

In the 7 years when all four prey variables were quantified (2009–2010, 2012, 2014–2017), correlations among all variables were positive with four of six being significant (*p* < 0.1), including the number of shoals with shoal area, shoal biomass, survey biomass, as well as survey biomass with shoal area (Figure 3). GAMMs examining the influence of spawning timing on each prey variable revealed a significant positive linear relationship between spawning timing and number of shoals along with a significant nonlinear relationship between spawning timing and shoal biomass (Table 2), with shoal biomass peaking within a week of spawning initiation (Figure 4). Spawning timing, however, was not significantly related to survey biomass or shoal area (Table 2; Figure 4). Indeed, survey biomass was consistently low until spawning began but became highly variable after spawning rather than consistently high (Figure 4).

**TABLE 2:** Generalized additive mixed models (GAMMs) showing the influence of the timing of capelin spawning (predictor) on prey variables (number of shoals, shoal area, shoal biomass, and survey biomass) during repeated surveys over a cluster of subtidal spawning sites of capelin on the northeast coast of Newfoundland, Canada during July–August, 2009–2010, 2012, 2014–2017.

Model	Days from start of spawning			
	edf	*F*	*p* Value	*r* ^2^
No. shoals	**1.00**	**28.3**	**<0.0001**	**0.13**
Shoal area	1.00	0.26	0.61	0.02
Shoal biomass	**2.97**	**5.58**	**0.003**	**0.12**
Survey biomass	2.08	1.57	0.25	0.04

Abbreviation: edf, estimated degree of freedom.

### 3.2 Seabird responses

**FIGURE 5:**
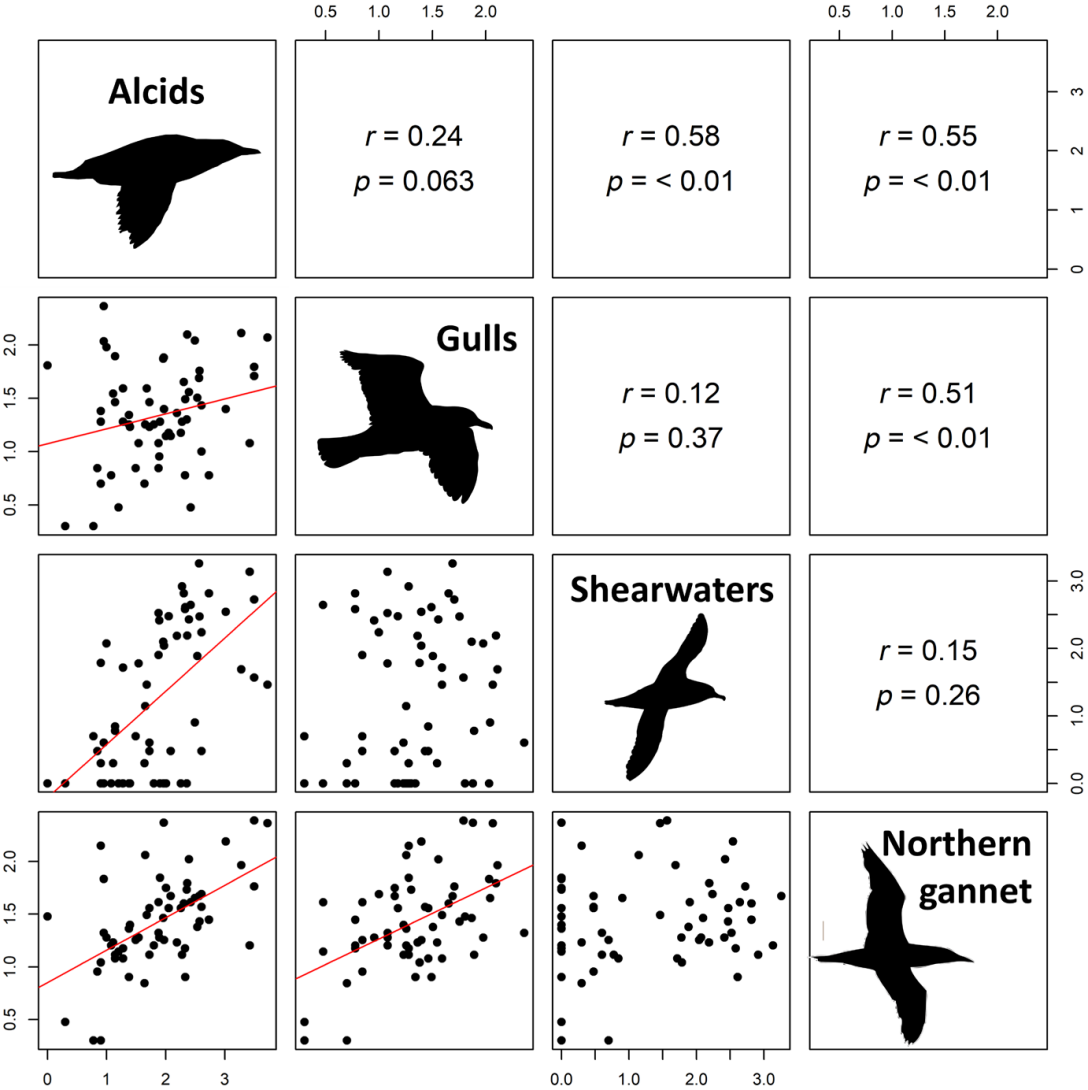
Pair‐wise relationships between the total number of birds within a species/group (alcids, gulls, shearwaters and Northern Gannets) per survey during repeated surveys over a cluster of subtidal spawning sites of capelin on the northeast coast of Newfoundland, Canada during July–August, 2009–2010, 2012, 2014–2020. Lower panels show relationships graphically, with red lines indicating significant relationships (*p* < 0.1) and upper panels show *r* and *p* values from correlation tests.

**FIGURE 6:**
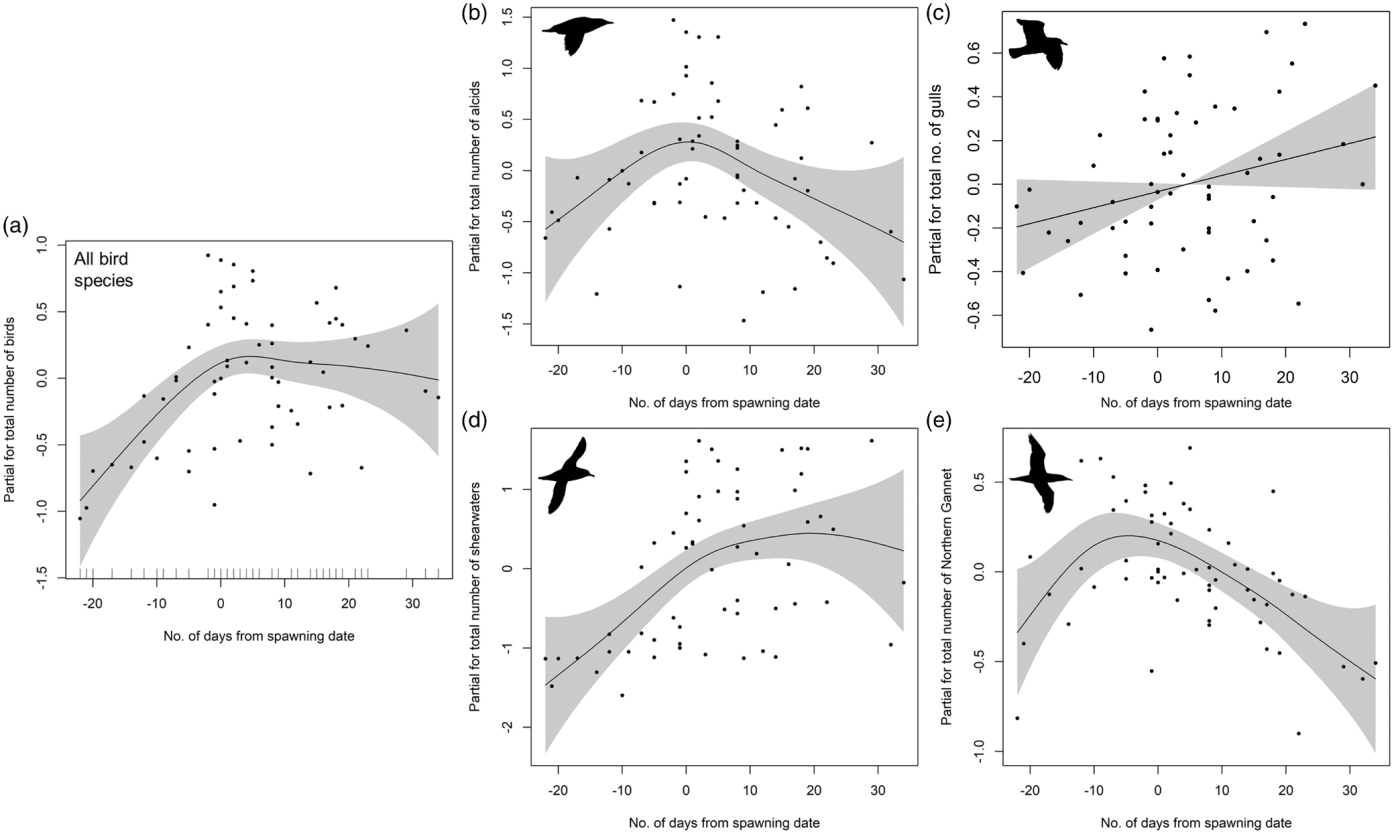
Estimated effect of the timing of capelin spawning (zero represents the first day of spawning in a particular year, while negative numbers represent days before spawning and positive numbers represent days after spawning) on (a) total number of birds (all species), (b) alcids (Razorbills, Common Murres, Atlantic Puffins), (c) gulls (American Herring Gull, Great Black‐backed Gull), (d) shearwaters (Great Shearwater, Sooty Shearwater) and (e) Northern Gannets on the northeast coast of Newfoundland, Canada during July–August, 2009–2010, 2012, 2014–2020. Grey area represents 95% confidence intervals.

The abundances of seabird species/groups within the survey area were significantly (*p* < 0.1) positively correlated (*R* values = 0.12–0.58), except for shearwaters with Northern Gannets and gulls (Figure 5). GAMMs examining the influence of survey biomass and spawning timing on seabird abundance revealed significant positive relationships of total birds along with each seabird species/group with spawning timing (*p* < 0.1) but not survey biomass (Table 3; Figure 6). The relationships between spawning timing and seabird abundance were primarily nonlinear and bell‐shaped, where the abundance of all seabirds, alcids, Northern Gannets and shearwaters peaked near the first day of spawning (Day 0) and decreased with the number of days before or after this date. By contrast, gull abundance increased linearly throughout the summer (Figure 6).

**TABLE 3:** Generalized additive mixed models (GAMMs) showing the influence of survey biomass and the timing of capelin spawning (predictors) on the total number of seabirds (all species), alcids (Razorbill, Common Murre, Atlantic Puffin), gulls (Herring Gull, Great Black‐backed Gull), Northern Gannets and shearwaters (Great Shearwater, Sooty Shearwater) on the northeast coast of Newfoundland, Canada during July–August, 2009–2010, 2012, 2014–2020.

Model	**Survey biomass**			**Days from start of spawning**			
	edf	*F*	*p* Value	edf	*F*	*p* Value	*r* ^2^
All bird species	1.86	1.76	0.26	**2.95**	**5.89**	**0.002**	**0.25**
Alcids	1.70	0.50	0.58	**2.92**	**3.64**	**0.030**	**0.15**
Gulls	1.56	0.69	0.33	**1.00**	**3.20**	**0.080**	**0.01**
Northern Gannet	2.23	2.55	0.09	**3.40**	**6.24**	**0.001**	**0.27**
Shearwaters	1.00	0.09	0.76	**2.48**	**7.27**	**0.001**	**0.16**

Abbreviation: edf, estimated degree of freedom.

**FIGURE 7:**
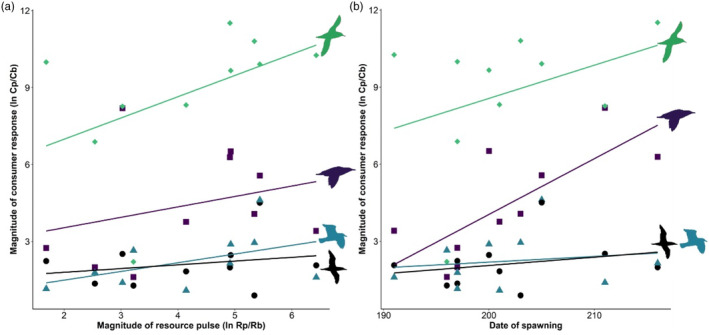
The magnitude of the consumer (seabird) response (abundance; ln Cp/Cb) relative to the (a) resource (capelin biomass) pulse magnitude (ln Rb/Rb) and (b) first date of spawning for each seabird species/group (alcids, gulls, shearwaters, Northern Gannets) during approximately weekly surveys over a cluster of subtidal spawning sites of capelin on the northeast coast of Newfoundland, Canada during July–August, 2009–2010, 2012, 2014–2020.

The resource pulse magnitude (ln Rp/Rb) ranged from 1.7 to 6.4 (Table 1), resulting in an average increase in prey biomass (Rp/Rb) due to the inshore migration of spawning capelin of 146 ± 59 times background levels (range: 5–619 times). The ANCOVA comparing the magnitude of the resource pulse (ln Rp/Rb) to the magnitude of the consumer response (ln Cp/Cb) revealed an overall significant positive relationship (slope: 0.430, *F*_1,32_ = 4.541, *p* = 0.041, *R* ^2^ = 0.747), with no difference in slopes among seabird species/groups, indicated by a nonsignificant interaction (*F*_3,32_ = 0.508, *p* = 0.679; Figure 7a). The mean adjusted response magnitude differed among seabird species/groups (*F*_3,32_ = 29.442, *p* < 0.0001), where the response magnitude of shearwaters was higher than alcids, gulls and Northern Gannets (*p* < 0.0001) and the response magnitude of alcids was higher than gulls (*p* = 0.011) and Northern Gannets (*p* = 0.008; Figure 7a). As the magnitude of the resource pulse (ln Rp/Rb) tended to be positively correlated with the date of peak survey biomass (*R* = 0.616, *n* = 10, *p* = 0.060), the ANCOVA examining the magnitude of the predator response (ln Cp/Cb) to the timing of the resource pulse (date of Rp) revealed a similar relationship to the magnitude of the resource pulse (ln Rp/Rb; results not shown). Given that seabirds appeared to respond primarily to spawning timing, rather than prey biomass (survey biomass), we examined whether the magnitude of the predator response (ln Cp/Cb) varied with spawning timing. An ANCOVA revealed an overall significant positive relationship (slope: 0.10, *F*_1,32_ = 6.871, *p* = 0.013, *R* ^2^ = 0.777; Figure 7b), with no difference in slopes of the relationships among seabird species/groups (interaction: *F*_3,32_ = 1.437, *p* = 0.250), and significant differences in the mean adjusted response magnitude among seabird species/groups (*F*_3,32_ = 33.400, *p* < 0.0001) were similar to the ANCOVA results reported for ln Rp/Rb versus ln Cp/Cb above (Figure 7b).

## 4 DISCUSSION

Our study adds to the growing literature illustrating temporary aggregative responses of multiple mobile predators to a pulsed resource, which has been shown across ecosystem types. Indeed, we showed that the inshore migration of spawning capelin acts as a pulsed resource, resulting in 5–619 times (146 ± 59 times) increase in coastal fish biomass. The magnitude of this resource pulse (ln Rp/Rb ~1.7–6.4) is similar to other studies in terrestrial, freshwater and marine ecosystems (ln Rp/Rb range: ~0.5–8, reviewed in Yang et al., 2010), as was the slope of the positive relationship (0.43) between predator aggregative response magnitude (ln Cp/Cb) and the resource pulse magnitude (slope range: ~0.4–1.0; Yang et al., 2010). As also commonly reported, the aggregative response of predators to this resource pulse led to structural changes in the predator assemblage (Ostfeld & Keesing, 2000; Yang et al., 2008, 2010), resulting from the influx of migratory species (non‐breeding shearwater species, Davoren, 2007, 2013a; humpback whales, Johnson & Davoren, 2021b) and increased abundance of breeding seabird species into our surveyed area. Overall, we showed that the marine ecosystem on the northeast Newfoundland coast follows previous definitions of a community altered by a pulsed resource (e.g., Ostfeld & Keesing, 2000; Yang et al., 2008, 2010).

The onset of this pulsed resource was associated with a shift in the coastal prey field toward more, larger and denser shoals. Despite the dramatic change in the prey field, shoal biomass was the only prey variable that peaked with spawning initiation (within the first week), likely due to the presence of large, persistent spawning shoals of capelin (Davoren et al., 2006). Interestingly, we did not find a significant relationship between fish biomass within the survey area (survey biomass) and spawning timing, likely due to the high variability in survey biomass after spawning began. This variation in survey biomass likely results from inter‐annual variation in the duration of spawning in the study area (~1–3 weeks; Crook et al., 2017; Penton et al., 2012), multiple spawning waves of capelin (waves separated by 6–14 days; Crook et al., 2017; Maxner et al., 2016; Penton et al., 2012; Tripp et al., 2023), and sex‐specific capelin spawning behaviour. To illustrate, male capelin arrive first and remain at spawning sites throughout spawning, while females complete maturation in nearby staging areas and then intermittently move toward spawning sites (Davoren, 2013b; Davoren et al., 2006; Templeman, 1948). Once spawning is complete, large spawning shoals dissipate, due to fish dying (mostly males) during spawning (Templeman, 1948) and smaller schools of survivors (mostly females) moving back offshore (Davoren, 2013b, Davoren et al., 2006). This sex‐specific behaviour may also explain the decrease in shoal biomass a few weeks after the initiation of spawning while the number of shoals continued to increase. Overall, this biology and behaviour of capelin likely resulted in inconsistently high fish biomass in the survey area after the initiation of spawning.

### 4.1 Predator responses to pulsed resource

The high variability in survey biomass after spawning began likely resulted in a lack of the predicted positive non‐linear relationship between fish biomass in the survey area (survey biomass) and seabird abundance. This finding is contrary to similar studies that found positive nonlinear, threshold relationships between marine predator and prey density (Cox et al., 2013; Marston et al., 2002; Piatt, 1990; Piatt et al., 2007; Piatt & Methven, 1992; Sigler et al., 2004) as well as others that found functional relationships between forage fish density and various parameters of seabird foraging behaviour and breeding success (e.g., Boyd & Murray, 2001; Cury et al., 2011; Guillemette et al., 2018; Harding et al., 2007; Mori & Boyd, 2004; Piatt et al., 2007; Womble et al., 2014). Alternatively, the abundance of all seabirds along with most seabird species/groups showed a bell‐shaped relationship with capelin spawning timing, suggesting that seabirds respond more to the seasonal influx of spawning capelin than short‐term fluctuations in average prey biomass in the survey area. In support, previous meso‐scale surveys in the larger study region found limited evidence for fish density explaining seabird density (Carvalho & Davoren, 2019; Davoren et al., 2003a, 2003b). Instead, seabirds are generally distributed in shallow (<50 m) regions along the coast, where they aggregate within finer scale areas (10 km^2^), including the survey area, that are characterized by the predictable presence of high abundance capelin aggregations once capelin arrive inshore to spawn (Davoren, 2007, 2013a; Davoren et al., 2003a, 2006). This aggregative response to the presence of spawning capelin rather than fish biomass might represent a better foraging strategy for predators, due to the variable prey biomass after spawning but generally higher number of larger, denser shoals in the survey area. Additionally, large, stationary spawning shoals of capelin at annually persistent and discrete spawning sites can be predictably located on a fine scale (<1 km) and capelin within these shoals do not elicit an escape response under predatory attacks (Davoren et al., 2006). Overall, fish biomass may be less important during selection of this foraging area by seabirds relative to the predictable presence of spawning shoals that allow high foraging efficiency (Sigler et al., 2017).

The lack of relationship between predator abundance and fish biomass may also result from seabirds using ‘short‐cut’ methods to indirectly track capelin biomass in the survey area rather than directly sampling. In support, at‐sea experiments within and nearby the survey area during years of this study (2009, 2013, 2015–2017) showed that seabirds are visually attracted to the presence of conspecific and heterospecific aggregations at sea (Bairos‐Novak et al., 2015; Carvalho et al., 2020; Maynard et al., 2019), as reported for seabirds in other regions (Tremblay et al., 2014; Thiebault, Mullers, Pistorius, & Tremblay, 2014; Thiebault, Mullers, Pistorius, Meza‐Torres, et al., 2014; reviewed in Monier, 2024). Meso‐scale surveys in the larger study region also often reveal tighter associations of seabirds with other marine predators than with fish (Carvalho & Davoren, 2019; Davoren et al., 2010). Significant positive correlations between seabird species/groups in most pairwise comparisons in this study further support that increased abundances of foraging seabirds in our survey area could act as cues to the presence of spawning capelin. As the survey area represents a cluster of annually persistent capelin spawning sites (Penton & Davoren, 2012), seabirds may also use memory to return to the survey area once capelin spawning begins (Davoren et al., 2003a). In support, seabirds in the study area are known to overfly aggregations of foraging predators during their commute to these spawning sites (Bairos‐Novak et al., 2015; Davoren et al., 2003a), as also observed in other regions (e.g. Irons, 1998). Additionally, seabirds breeding nearby the survey area regularly exhibit directional flight to these areas to forage, based on tracking studies of Northern Gannets (Garthe et al., 2007, 2011; Montevecchi et al., 2009), Common Murres (Gulka et al., 2020; Gulka & Davoren, 2019; Montevecchi et al., 2019), Razorbills (Gulka et al., 2019; Lescure et al., 2023) and Great Black‐backed Gulls (Maynard et al., 2021). Using memory‐based foraging may result in high seabird abundance within the survey area as prey biomass declines until this behaviour is extinguished (Davoren et al., 2003a), which is supported by the gradual decline in seabird abundance later in the season.

Although more, high‐density fish shoals could be predictably located within the survey area during capelin spawning in all years, the magnitude of the resource pulse varied almost fourfold (1.7–6.4) across the 10 years of this study. As demonstrated previously in a variety of ecosystems (Yang et al., 2010), the magnitude of the predator aggregative response increased with the magnitude of the resource pulse (Yang et al., 2010). In our study, this relationship may result from several factors. First, lower magnitude pulses may go unnoticed by predators because the increase in prey biomass cannot be distinguished as being above the average of the environment (prey patch selection theory; Stephens & Krebs, 1986). This inability to perceive low‐magnitude pulses may be exacerbated if predators use indirect methods to track prey (e.g. local enhancement, memory). Second, seabirds may reduce dietary reliance on capelin under low‐magnitude resource pulse years, as shown previously for multiple predators (Carvalho & Davoren, 2020; Gulka et al., 2020; Lescure, 2021). Third, as patch profitability may be related to both prey and competitor densities (Fretwell & Lucas, 1969), some seabird species may avoid foraging under higher competitor densities during years of low‐magnitude resource pulses. In support, a numerically dominant seabird in the study area, the Common Murre, tends toward uniform spacing at fine spatial scales (~250 m) within the survey area (Davoren et al., 2003b), dives solitarily to capture individual capelin that become separated from shoals (Crook & Davoren, 2014), and direct (e.g. food stealing) and indirect (e.g. displacement) interactions among other seabird species during prey capture are higher under lower capelin biomass conditions (Carvalho et al., 2020; Maynard et al., 2019).

Although we predicted a lower magnitude response by predators when the resource pulse was temporally unpredictable (Willson & Womble, 2006; Yang et al., 2010), a higher magnitude predator response was observed when the resource pulse was temporally delayed from the long‐term average (Day 199, or July 18) during the later part of the time series (i.e. 2017–2020). This delayed resource pulse resulted in low fish biomass during energetically demanding periods for seabirds (i.e. moulting for non‐breeding shearwaters, Carvalho et al., 2022; chick‐rearing for breeding seabirds; Davoren, 2024). Therefore, this higher magnitude response during years of delayed capelin spawning may result from the urgency of seabirds to maximize encounter rates with prey during these energetically demanding stages of their annual cycle. In support, seabird foraging effort during chick‐rearing is much higher in years when capelin arrival in the study area is delayed (Common Murres, Gulka et al., 2020; Gulka & Davoren, 2019; Montevecchi et al., 2019; Razorbills, Lescure et al., 2023). These higher magnitude responses may allow seabirds to maintain breeding success while ensuring their own survival, as predicted for long‐lived seabirds (Stearns, 1992). If parental energy expenditure exceeds physiological limits, however, parents might not be able to buffer the temporal mismatch between this resource pulse and seabird breeding, resulting in reduced chick provisioning rates and reproductive success (Carscadden et al., 2002; Regular et al., 2014).

### 4.2 Guild‐specific predator responses

Seabird responses to the shift in the prey field appeared to be guild‐specific and differences are likely related to dietary tendencies (e.g. specialists, generalist) and breeding status (e.g. breeding, non‐breeding). Non‐breeding shearwaters had the highest magnitude response to the pulsed resource, possibly due to their high dietary reliance on capelin in coastal Newfoundland (Carvalho et al., 2022; Carvalho & Davoren, 2020; Gulka et al., 2017), tendency to feed within the survey area (Davoren, 2013a) and lack of spatial constraints to forage within range of local breeding colonies. Their own migratory schedules (Hedd et al., 2012) and/or a lack of knowledge of inshore capelin biomass may explain the higher time lag between the peak abundance of shearwaters and the first day of spawning compared to breeding species, as shown for other non‐breeding, migratory predators (humpback whales; Johnson & Davoren, 2021b). By contrast, spatial constraints to forage within limits of breeding colonies and the high dietary reliance on capelin by the numerically dominant species of breeding alcid (Common Murre; Davoren & Montevecchi, 2003; Montevecchi et al., 2019) likely explain the minimal time lag between the peak abundance of alcids and the first day of spawning, along with the higher magnitude response of alcids relative to other breeding seabirds (Northern Gannets, gulls). The steep decline in abundance of alcids later in the summer likely results from parents and offspring departing the study area post‐breeding in mid‐August (Runnells et al., 2024). Northern Gannet abundance showed a similar bell‐shaped curve to alcids but had a lower magnitude response, likely due to their lower dietary reliance on capelin in the study area (Montevecchi, 2007) and tendency to forage outside of the survey area (Davoren, 2013a; Garthe et al., 2007, 2011). Similarly, large gulls in the study area consume a high diversity of prey types (Maynard & Davoren, 2020) and often forage outside of the survey area (Maynard et al., 2021). The continued increase in gull abundance and lack of a steep decline in shearwater abundance in the survey area later in the season may be related to the increase in other available prey types. In particular, Atlantic cod (*Gadus morhua*) is commercially fished within the survey area (Davoren, 2007) from early August to September, and adult and juvenile gulls along with non‐breeding shearwaters are known to aggregate in high abundances at fishing boats discarding cod offal at sea (Carvalho et al., 2020, Maynard et al., 2019). Overall, dietary specialization on capelin appeared to result in higher magnitude responses to the pulsed resource relative to species with more generalist tendencies, suggesting that varying timing and magnitude of the pulse may have a greater impact on capelin specialists.

## 5 CONCLUSIONS

As pulsed resources provide natural experimental conditions to study both predator species‐level and community‐level responses to resource variability, this and other similar studies provide insight into the ability of natural systems to tolerate natural and human‐induced disturbance events (Yang et al., 2008, 2010). In marine systems, exploring these predator–prey relationships may inform precautionary harvest rules for forage fish (Pikitch et al., 2012) by detecting the minimum fish biomass required for predators to aggregate in an area (Piatt, 1990; Piatt & Methven, 1992). Our findings, however, suggest that minimum prey thresholds may be difficult to detect when forage fish act as a pulsed resource and aggregate predictably in time and space. Indeed, predators did not respond to short‐term changes in prey biomass within this predictable foraging area; they, instead, aggregated within these areas based on the binary presence (vs. absence) of a pulsed resource. Therefore, predator responses to region‐wide declines in prey biomass may go undetected, in contrast to findings of previous studies (e.g. Cury et al., 2011; Harding et al., 2007; Piatt et al., 2007). Indeed, seabirds and other predators in coastal Newfoundland continue to aggregate in response to this influx of spawning capelin as they did before the stock collapsed in the early 1990s, despite the persistent 30‐fold decrease in capelin biomass on the Newfoundland Shelf (Buren et al., 2019). Across years, however, variation in the timing and magnitude of this pulsed resource within this predictable foraging area resulted in altered seabird aggregative responses (this study), diet (Carvalho & Davoren, 2020; Gulka et al., 2020; Lescure, 2021) and foraging effort (Garthe et al., 2011; Gulka et al., 2020; Gulka & Davoren, 2019; Lescure et al., 2023; Montevecchi et al., 2019). These findings reiterate the need for long‐term studies that simultaneously quantify responses of multiple predator species to prey biomass to assess the impact of varying prey regimes on predator population dynamics (Cury et al., 2011). As increasing human exploitation of forage fish species may impact predator population dynamics (Cury et al., 2011; Essington et al., 2015; Pikitch et al., 2012), these long‐term studies will continue to inform ecosystem‐based fisheries management on a global scale. Fisheries will also interact with climate change to alter forage fish population dynamics. For Newfoundland capelin, sea ice extent and spring sea ice retreat timing appear to shape spawning timing and biomass (Buren et al., 2014) and climate change projections for the Newfoundland Shelf (Han et al., 2015) suggest lower magnitude and delayed timing of inshore capelin pulses in the future. These changes combined with other climate‐driven changes, such as variable capelin quality (e.g. energy density) and competitive interactions with capelin‐dependent ectothermic predators (e.g. Atlantic cod) and other endothermic predators (e.g. baleen whales), may result in negative consequences for seabird populations (Piatt et al., 2020).

## AUTHOR CONTRIBUTIONS

Gail K. Davoren conceived the ideas, designed methodology, acquired funding, collected data, processed data, analysed data, interpreted data and led the writing of the manuscript. Laurie D. Maynard collected data and analysed data. Kelsey F. Johnson, Paloma C. Carvalho, Julia Gulka, Edward Jenkins, Lauren M. Lescure, Emily Runnells and Ashley Tripp collected data and processed data. All authors contributed critically to drafts and approved the final version for publication.

## CONFLICT OF INTEREST STATEMENT

The authors declare there are no competing interests.

## STATEMENT ON INCLUSION

Our study brings together authors from several different countries, including scientists based in the country where the study was carried out. All authors were engaged early on with the research and study design and throughout the research to ensure that the diverse sets of perspectives they represent was considered from the onset. Whenever relevant, literature published by scientists from the region was cited. We shared research outcomes with local stakeholder groups (Newfoundland fishers) within the study region through informal talks, presentations and social media posts.

## Data Availability

Data available from the Dryad Digital Repository https://doi.org/10.5061/dryad.jq2bvq8k8 (Davoren et al., 2024).
